# Revisiting TREM2: from multi-omics signaling networks to clinical translation

**DOI:** 10.3389/fimmu.2026.1782384

**Published:** 2026-07-28

**Authors:** Jing Yang, Dehong Wan, Lina Ren, Yaqing Lv, Hai Zhao

**Affiliations:** 1 Department of Emergency Surgery, The Affiliated Hospital of Qingdao University, Qingdao, Shandong, China; 2 Department of Operating Room, The Affiliated Hospital of Qingdao University, Qingdao, Shandong, China; 3 Department of Neurosurgery, The Affiliated Hospital of Qingdao University, Qingdao, Shandong, China

**Keywords:** Alzheimer’s disease, cancer immunotherapy, microglia, neuroinflammation, systems biology, TREM2, Tumor-associated macrophages

## Abstract

TREM2 is a cell surface receptor that plays a crucial role in regulating immune responses, particularly in myeloid cells such as macrophages, dendritic cells, and microglia. Initially studied for its involvement in neuroinflammation and neurodegenerative diseases, while recent evidence has highlighted its broader implications in cancer and various other diseases. Recent studies show that TREM2 plays an immunoprotective role prompted by central nervous system-enriched sphingolipids during GBM progression. TREM2 overexpression represses GBM and synergizes with anti-PD-1 therapy, suggesting a potential therapeutic avenue in cancer immunotherapy. This review provides a comprehensive overview of TREM2 biology, focusing on its molecular structure, signaling pathways, and functional roles in different disease contexts. In Alzheimer’s disease, TREM2 mutations are associated with altered immune responses and impaired amyloid-beta clearance, suggesting a potential therapeutic target. In cancer, TREM2 contributes to immune evasion, tumor progression, and metastasis, with emerging strategies aiming to target TREM2 to enhance anti-tumor immunity. Additionally, TREM2 is implicated in a range of other conditions, including autoimmune diseases, multiple sclerosis, and Parkinson’s disease, where it may regulate inflammatory responses and tissue damage. Despite promising findings, several challenges remain in understanding TREM2’s complex role across diverse diseases. Future research directions include unraveling its precise mechanisms, validating its therapeutic potential, and exploring clinical interventions targeting TREM2. This review provides a comprehensive overview of TREM2 biology, synthesizing insights from recent single-cell transcriptomics and metabolic profiling. We focus on its molecular structure, complex signaling networks, and context-dependent functional roles across diverse disease landscapes.

## Introduction

TREM2 (Triggering Receptor Expressed on Myeloid cells 2) is a cell surface receptor predominantly expressed on myeloid cells, including macrophages, dendritic cells, and microglia, and plays a pivotal role in modulating immune responses (1). As a member of the immunoglobulin superfamily, TREM2 is involved in a variety of cellular functions, including phagocytosis, immune cell activation, and the regulation of inflammatory responses (1). Initially identified for its expression in the CNS (central nervous system) and its involvement in neuroinflammatory conditions, TREM2 has since emerged as a critical modulator in a wide range of disease processes, spanning neurodegenerative diseases (NDDs), cancer, autoimmune disorders, and inflammatory conditions (2–4).

The most extensively studied role of TREM2 has been in the context of Alzheimer’s disease (AD), where genetic variants of TREM2 are associated with an increased susceptibility to the disease (5–7). Mutations in TREM2, particularly the R47H variant, are known to impair microglial function and increase AD risk, as detailed in Section 4. Recent studies have suggested that TREM2 may also play a neuroprotective role by modulating microglial activity, which has been linked to the regulation of neurodegenerative processes (2, 8). These insights have positioned TREM2 as a promising therapeutic target in the development of immunomodulatory therapies aimed at mitigating neuroinflammation and enhancing the clearance of amyloid plaques in AD patients (8, 9).

However, TREM2’s biological function is not confined to NDDs (10). Recent evidence has broadened the scope of its relevance, particularly in the realm of cancer (11–14). Tumor-associated myeloid cells, such as macrophages, are critical players in shaping the tumor microenvironment (TME), influencing immune evasion, tumor progression, and metastasis (15, 16). In this context, TREM2 has emerged as a key modulator of macrophage polarization, particularly in the regulation of tumor-associated macrophages (TAMs) (17). TREM2 expression on TAMs has been shown to facilitate the immune suppressive phenotype of these cells, which in turn promotes tumor growth and metastasis. Notably, TREM2 has been implicated in the response to immunotherapy, with studies demonstrating that TREM2 overexpression can suppress tumor progression in models of glioblastoma (GBM) and enhance the efficacy of immune checkpoint inhibitors, such as anti-PD-1 therapy (3). This suggests that TREM2 may not only contribute to immune evasion in cancer but also serve as a novel therapeutic target to augment the effectiveness of existing immunotherapies.

In addition to its roles in AD and cancer, TREM2 is also implicated in a variety of other diseases, ranging from autoimmune disorders to infectious diseases (18, 19). In conditions such as rheumatoid arthritis and multiple sclerosis, TREM2 appears to modulate inflammatory pathways by influencing the activation and function of myeloid cells, including macrophages and dendritic cells (20–22). By regulating the balance between pro-inflammatory and anti-inflammatory signals, TREM2 may play a critical role in tissue damage and repair processes (23). Moreover, recent studies have suggested that TREM2 could be involved in the resolution of inflammation in conditions like sepsis, where the receptor’s expression on myeloid cells aids in controlling excessive immune responses and promoting tissue repair (23).

Despite the growing body of evidence highlighting TREM2’s role in various diseases, several questions remain unanswered regarding its precise mechanisms of action, its interaction with other immune signaling pathways, and its potential as a therapeutic target. Furthermore, while TREM2 has been shown to exert beneficial effects in certain disease contexts, its role in immune suppression in cancer and chronic inflammation warrants careful consideration. As research continues to elucidate the diverse functions of TREM2 across different diseases, it is becoming increasingly clear that this receptor holds significant potential not only as a biomarker but also as a therapeutic target in precision medicine.

Crucially, our understanding of TREM2 has been revolutionized by systems biology approaches. The application of high-dimensional single-cell RNA sequencing (scRNA-seq) and spatial transcriptomics has moved the field beyond linear signaling models, identifying TREM2 as the master driver of the “Disease-Associated Microglia” (DAM) transcriptional signature. Furthermore, the integration of lipidomics with proteomic networks has revealed how TREM2 couples metabolic sensing to immune checkpoints.

Therefore, this review aims to provide a comprehensive overview of TREM2 by integrating these multi-omics insights. We focus on its biological functions, regulatory signaling networks, and its evolving dichotomy in Alzheimer’s disease versus cancer.

## Biology of TREM2

**Figure 1:**
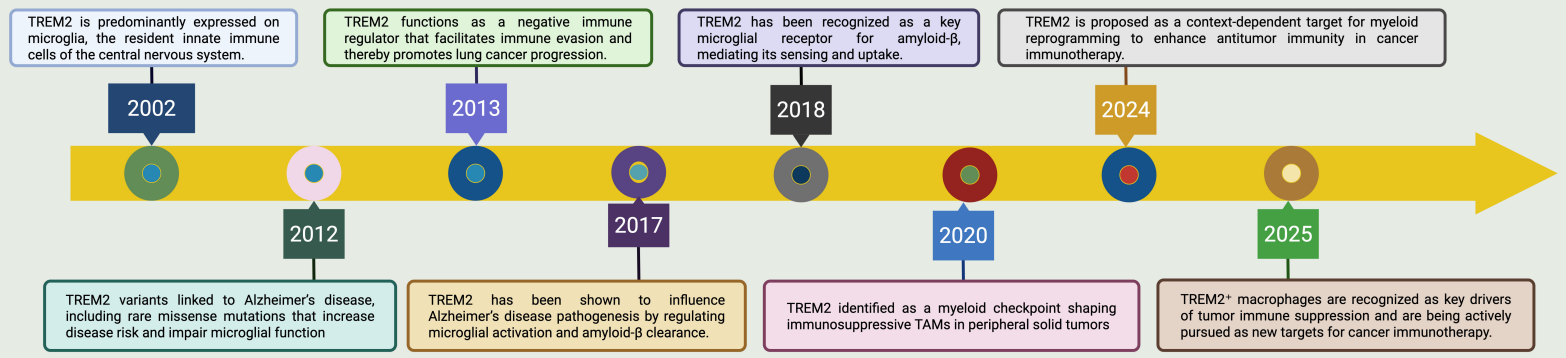
Key milestones in TREM2 research across neurodegeneration and cancer. Initially characterized as a key microglial molecule, TREM2 was later found to carry rare missense variants that markedly increase the risk of AD by impairing microglial function, amyloid-β sensing, and plaque clearance. Subsequent work in oncology revealed that TREM2 can also act as a negative immune regulator in lung cancer and a myeloid checkpoint that shapes immunosuppressive TAMs in peripheral solid tumors. These insights have led to the current view of TREM2 as a context-dependent, therapeutically actionable node, with TREM2^+^ macrophages recognized as major drivers of tumor immune suppression and active targets for myeloid-reprogramming strategies in cancer immunotherapy.

TREM2 is a transmembrane receptor that plays a crucial role in the regulation of innate immune responses. It was first identified in 2000 as a cell surface receptor primarily expressed on myeloid cells such as macrophages, dendritic cells, and microglia (24–26) (**Figure 1**). TREM2 belongs to the immunoglobulin superfamily, and its functional significance was initially linked to its role in microglial activation and neuroinflammation. Over the past two decades, research has expanded the understanding of TREM2’s functions beyond the nervous system, demonstrating its involvement in a variety of diseases, including Alzheimer’s disease, cancer, autoimmune disorders, and infections (27, 28).

### Structural features of TREM2

The TREM2 protein consists of three major structural domains: an extracellular immunoglobulin-like domain, a single transmembrane domain, and a short intracellular cytoplasmic tail (5). The extracellular portion of TREM2 contains a single immunoglobulin-like V-type (IgV) domain responsible for ligand binding (e.g., lipidated molecules, apoptotic cell components) (5). This structural feature distinguishes TREM2 from certain other immunoglobulin superfamily receptors (e.g., TREM1) that possess both IgV and IgC domains (24). These domains enable TREM2 to interact with a range of ligands, including anionic lipids, apolipoproteins (such as ApoE and ApoJ), and phosphatidylserine exposed on apoptotic cells, as demonstrated in microglial studies (1, 29). In tumor macrophages, lipid-rich necrotic debris may act as TREM2 agonists, engaging the same immunoglobulin-like V-type domain to trigger downstream DAP12–SYK signaling (3, 12, 30). The structure of the extracellular domain has been shown to be critical for the receptor’s function in recognizing and binding to these molecules, a key aspect of its role in immune modulation.

**Figure 2:**
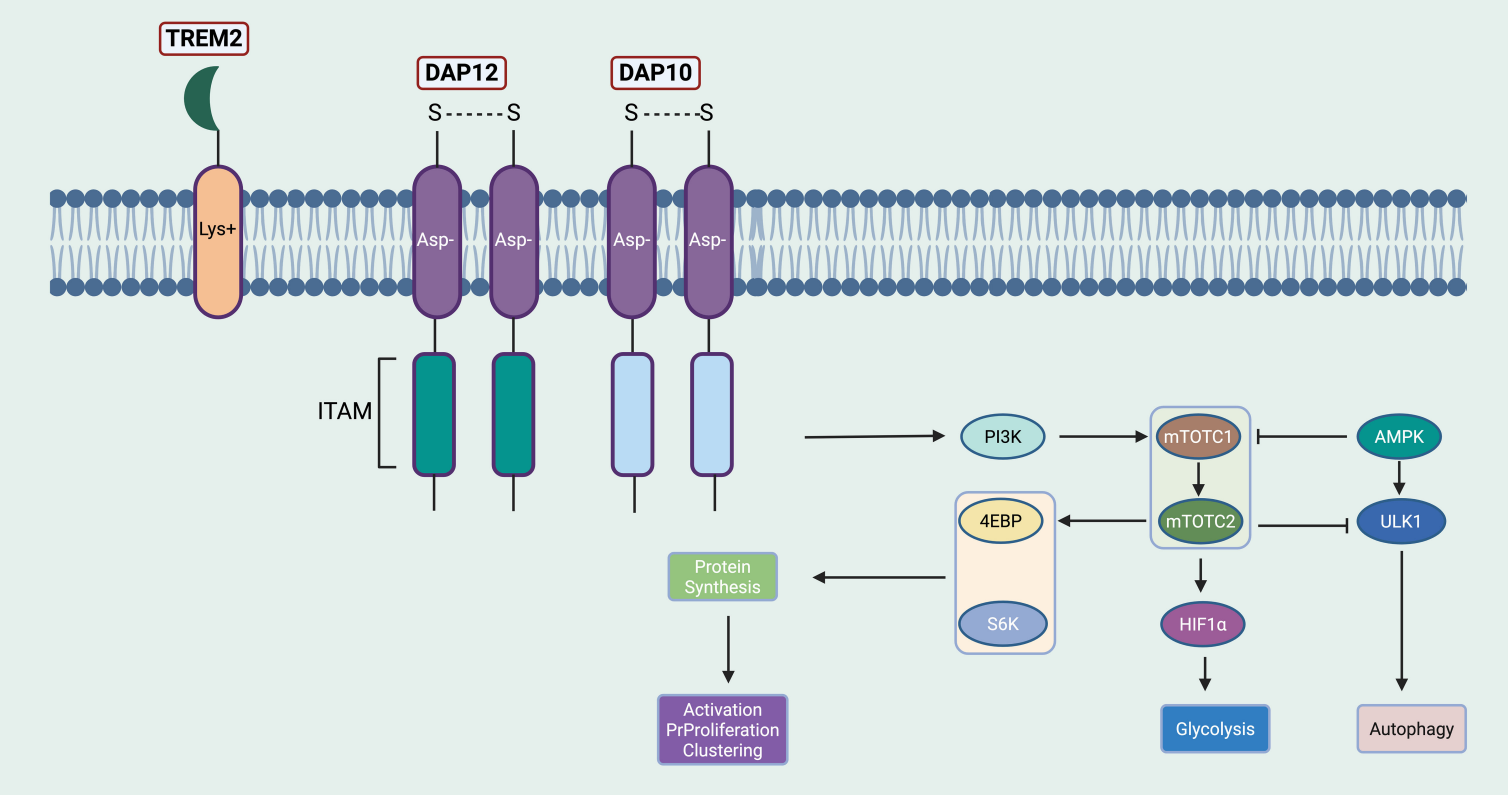
Structural coupling of TREM2 to DAP12/DAP10 and downstream PI3K–mTOR signaling. Upon ligand binding, TREM2 forms a signaling complex with the adaptor proteins DAP12 and DAP10 through electrostatic interactions between a positively charged lysine (Lys^+^) residue within the TREM2 transmembrane segment and negatively charged aspartate (Asp^-^) residues in the adaptor chains. DAP12 transduces intracellular signals via its ITAMs, which recruit and activate SYK, leading to downstream cascades that regulate cytoskeletal rearrangement, phagocytosis, and metabolic reprogramming. DAP10, in parallel, signals through its YINM motif, engaging PI3K and subsequently activating the PI3K/Akt/mTORC1/2 pathway. PI3K-mediated mTORC1 activation promotes phosphorylation of 4EBP and S6K, facilitating protein synthesis and cellular programs such as activation, proliferation, and clustering, whereas mTORC2 contributes to HIF-1α stabilization and enhanced glycolytic metabolism. The AMPK pathway antagonizes mTORC1 activity and, through ULK1, promotes autophagy to maintain metabolic balance. In the central nervous system, DAP12/SYK-dependent signaling in microglia promotes phagocytosis, lipid metabolism, and inflammatory resolution, whereas in the tumor microenvironment, particularly within tumor-associated macrophages, the same pathway supports anti-inflammatory polarization, tissue remodeling, and immune suppression. Thus, the cellular context—microglia versus tumor macrophages—critically determines whether TREM2 signaling results in immune activation or inhibition. Disulfide bridges are indicated as S—S. AMP, activated protein kinase; Asp^-^, Aspartate (negatively charged residue); DAP10, DNAX-activating protein of 10 kDa; DAP12, DNAX-activating protein of 12 kDa; ERK, Extracellular signal-regulated kinase; 4EBP, Eukaryotic translation initiation factor 4E-binding protein; HIF-1α, Hypoxia-inducible factor 1-alpha; IL-17, Interleukin-17; ITAM, Immunoreceptor tyrosine-based activation motif; Lys^+^, Lysine (positively charged residue); mTORC1/2, Mechanistic target of rapamycin complex 1/2; PI3K, Phosphatidylinositol-3-kinase; S6K, Ribosomal protein S6 kinase; sTREM2, Soluble form of triggering receptor expressed on myeloid cells 2; SYK, Spleen tyrosine kinase; TREM2, Triggering receptor expressed on myeloid cells 2; ULK1, Unc-51-like autophagy activating kinase 1; YINM, Tyrosine-Isoleucine-Asparagine-Methionine signaling motif.

The transmembrane domain anchors TREM2 in the cell membrane, while the cytoplasmic tail, which is relatively short, contains the signaling motifs necessary for intracellular signal transduction. Unlike many other immune receptors, the TREM2 cytoplasmic tail does not contain a classical immunoreceptor tyrosine-based activation motif (ITAM) (31). Instead, its signaling is primarily mediated by the adapter protein DAP12, which contains an ITAM that becomes phosphorylated upon TREM2 activation (32). This phosphorylation event triggers downstream signaling pathways, particularly the PI3K/Akt pathway, which promotes cell survival, immune modulation, and anti-inflammatory responses (32) (**Figure 2**). While membrane TREM2 primarily mediates DAP12-dependent intracellular signaling, sTREM2 (Soluble TREM2) functions as a paracrine modulator enhancing microglial survival and cytokine production (33, 34). However, in the tumor microenvironment, the relative abundance of sTREM2 versus membrane TREM2 on macrophages remains underexplored, though elevated sTREM2 has been linked to immunosuppressive myeloid reprogramming (35).

### Soluble TREM2: from proteolytic cleavage to paracrine signaling

While membrane-bound TREM2 acts as a receptor, its ectodomain can be proteolytically shed to generate sTREM2, a biologically active molecule that regulates neuroimmune homeostasis independent of the cell-autonomous signaling of the full-length receptor. This shedding is primarily mediated by the metalloproteases ADAM10 and ADAM17, which cleave the TREM2 stalk region at the H157–S158 peptide bond (36, 37). This process is not merely a degradation pathway but a regulated mechanism that dictates the balance between receptor signaling density and soluble factor availability.

Unlike a passive bystander, sTREM2 functions as a potent paracrine signaling molecule (38). Upon release into the extracellular space, sTREM2 can engage unrecognized receptors on neighboring microglia to promote cell survival and proliferation. Mechanistically, sTREM2 has been shown to activate the NF-κB pathway and induce the expression of pro-inflammatory cytokines, thereby boosting the innate immune alertness of the local microenvironment (7). This “priming” effect suggests that sTREM2 may act as a “danger signal” or cytokine-like factor that amplifies the microglial response to pathology. Furthermore, sTREM2 retains the capacity to bind amyloid-beta (Aβ) oligomers (39). By sequestering soluble Aβ aggregates, sTREM2 may prevent their assembly into neurotoxic plaques, effectively acting as a “decoy receptor” or chaperone that facilitates the clearance of pathological proteins.

The clinical utility of sTREM2 lies in its dynamic fluctuation across disease stages. In CSF, sTREM2 levels are often elevated in the early, asymptomatic stages of AD, correlating with markers of neuronal injury rather than amyloid burden alone. This elevation is interpreted as a compensatory response where microglia ramp up TREM2 expression and shedding to contain nascent damage. Longitudinal studies indicate that higher baseline sTREM2 levels are associated with a slower rate of cognitive decline and gray matter atrophy, reinforcing its protective role. Similarly, in amyotrophic lateral sclerosis (ALS), elevated CSF sTREM2 correlates with prolonged survival, serving as a prognostic marker of microglial activation (40).

The biology of sTREM2 presents a therapeutic paradox (41). While sTREM2 itself has protective properties, its generation comes at the cost of reducing surface TREM2, which is essential for DAP12-mediated phagocytosis and metabolic fitness. The TREM2 H157Y variant, for instance, increases shedding and reduces surface receptor levels, leading to functional haploinsufficiency. Therefore, therapeutic strategies targeting the “sheddase” machinerymust carefully balance the preservation of surface receptor signaling with the maintenance of beneficial sTREM2 levels.

### A systems biology perspective: integrating multi-omics networks

While classical signaling models depict TREM2 as a linear transducer of DAP12-phosphorylation events, a true systems biology perspective reveals that TREM2 functions as a master regulator within a complex, non-linear gene regulatory network (GRN) (42). The advent of high-dimensional multi-omics technologies—spanning single-cell transcriptomics, spatial profiling, and lipidomics—has fundamentally redefined our understanding of how TREM2 orchestrates myeloid cell identity and metabolic adaptation (43, 44). Rather than acting in isolation, TREM2 integrates metabolic inputs with transcriptional reprogramming to define distinct cellular states.

The Transcriptional Landscape: From Homeostasis to DAM Single-cell RNA sequencing (scRNA-seq) has provided the most definitive evidence of TREM2’s role as a transcriptional switch (45, 46). In the context of neurodegeneration, unbiased clustering of microglial populations identified a specific, conserved transcriptional state termed DAM or the “Microglial Neurodegenerative Phenotype” (MGnD) (8, 47, 48). Systems-level analysis of microglial trajectories reveals a two-step activation process. The first step, which involves the downregulation of homeostatic checkpoint genes (e.g., P2ry12, Tmem119, Cx3cr1), is TREM2-independent (49, 50). However, the second and critical step—the full acquisition of the DAM signature characterized by the upregulation of lipid-metabolism and phagocytic genes (e.g., Lpl, Apoe, Cst7, Clec7a, Spp1)—is strictly TREM2-dependent (30).

This transcriptional dependency highlights TREM2 not merely as a receptor but as a node that sustains a specific chromatin state required for the containment of pathology. In the absence of TREM2, microglia are transcriptionally “locked” in an intermediate, dysfunctional state, unable to mount the robust phagocytic program necessary for amyloid plaque compaction or myelin debris clearance. This defines TREM2 as a “state-change” regulator within the microglial GRN (46).

Spatial Transcriptomics and the “Niche” Effect: Biological systems are spatially organized, and TREM2 signaling is highly context-dependent. Spatial transcriptomics has added a geographical layer to this system, demonstrating that TREM2-enriched DAM populations are not randomly distributed but are spatially restricted to specific pathological niches, such as the immediate vicinity of amyloid-beta plaques in Alzheimer’s disease or the peri-necrotic zones in GBM.

This “niche-dependent” expression profile suggests a localized feedback loop: local ligands (e.g., exposed lipids on plaques or necrotic debris) trigger TREM2 clustering, which locally drives the expression of chemotactic and survival factors (51). This spatial confinement creates a “protective barrier” system, preventing the diffusion of neurotoxic oligomers. In contrast, in peripheral tumors, the spatial enrichment of TREM2+ macrophages in the tumor core correlates with regions of T-cell exclusion, suggesting that the TREM2 regulatory network actively remodels the local chemokine landscape to create an immunosuppressive niche (14).

Lipidomics and Metabolic Flux Networks At the metabolic level, systems biology integration of lipidomics and transcriptomics has unveiled TREM2 as a critical sensor of the brain’s lipid landscape. TREM2 does not simply bind lipids; it orchestrates a metabolic flux network that allows microglia to consume and process massive lipid loads that would otherwise be lipotoxic. TREM2-deficient cells exhibit a “metabolic gridlock,” characterized by the accumulation of cholesteryl esters and defective myelin turnover (52).

This metabolic failure is linked to the mTOR signaling hub (53). Multi-omics data indicate that TREM2 activation rewires the cellular bioenergetic network, shifting microglia from a quiescent state to a high-energy state utilizing glycolysis and fatty acid oxidation (FAO) (54–56). This shift is essential to fuel the high energy demands of phagocytosis and cytoskeletal remodeling. Thus, from a systems perspective, TREM2 couples “sensing” (ligand binding) with “fueling” (mTOR-mediated metabolic shift), ensuring that the transcriptional demand for phagocytosis is matched by metabolic supply (57).

Proteomics and the Soluble Interactome Finally, the proteomic dimension adds complexity through the generation of soluble sTREM2. Proteomic screens suggest that sTREM2 is not merely a degradation byproduct but an active component of the intercellular signaling network (58, 59). Elevated levels of sTREM2 in CSF correlate with specific proteomic clusters associated with inflammation and neuronal injury (60). This suggests a paracrine feed-forward loop where sTREM2 may act to propagate survival signals or modulate synaptic function beyond the immediate cell surface.

In conclusion, a systems biology approach integrates these layers—transcriptional switches, spatial niches, metabolic flux, and proteomic networks—to frame TREM2 as a central hub in the “immune-metabolic interface.” It is this multi-layered connectivity that explains its dualistic nature: protective when resolving specific lipid pathologies in the CNS, yet detrimental when fueling immunosuppressive metabolism in the tumor microenvironment.

### Biological functions of TREM2

Upon ligand binding, TREM2 activates several intracellular signaling cascades that mediate various biological functions (61). One of the key roles of TREM2 is in regulating macrophage and microglial activation (6, 62). In microglia, TREM2 activation leads to the enhancement of phagocytosis, particularly in the clearance of apoptotic cells and debris, which is critical for maintaining tissue homeostasis and preventing chronic inflammation (63, 64). This function is especially important in the context of NDDs, where defective clearance of neurotoxic substances like amyloid-beta plaques in Alzheimer’s disease (AD) contributes to disease progression (65, 66).

**Figure 3:**
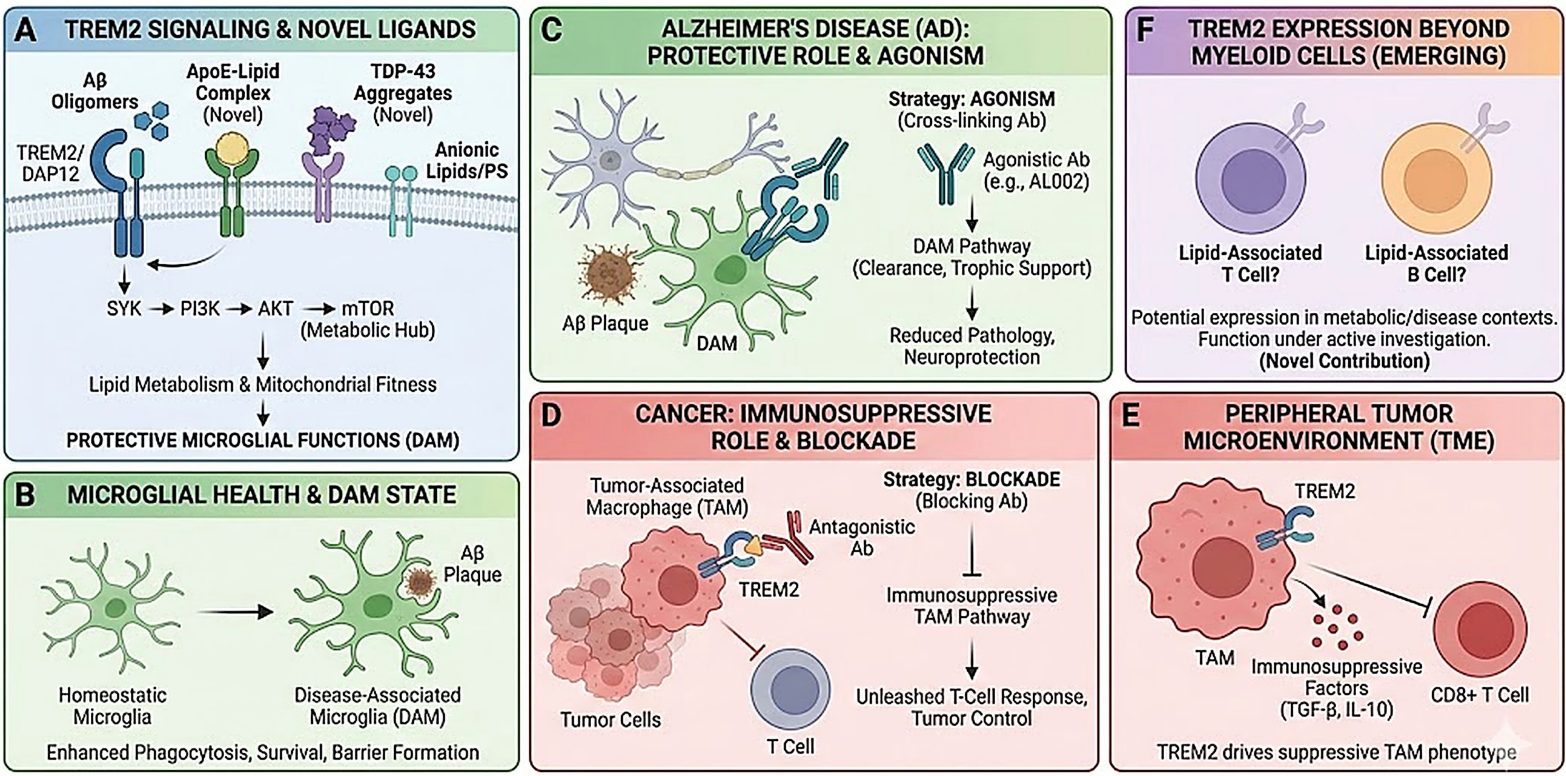
The evolving landscape of TREM2 biology: context-dependent roles and emerging frontiers. This schematic summarizes recent advances defining how triggering receptor expressed on myeloid cells 2 (TREM2) integrates lipid-sensing with myeloid-cell reprogramming across neurodegeneration and cancer, and highlights emerging non-canonical expression patterns. **(A)** TREM2 signaling and expanding ligand space. TREM2, in complex with adaptor proteins (classically DAP12, and in some contexts DAP10), transduces extracellular cues—including Aβ–associated species, ApoE–lipid complexes, anionic phospholipids such as phosphatidylserine (PS), and other damage-associated lipidated aggregates—into intracellular activation programs. Ligand engagement initiates signaling through SYK and PI3K–AKT–mTOR nodes, linking receptor activation to metabolic fitness (lipid handling, mitochondrial function) and immune effector remodeling. **(B)** Microglial homeostasis and the disease-associated microglia (DAM) transition. Under homeostatic conditions, microglia maintain surveillance and barrier functions; with pathology, TREM2-dependent programs promote a DAM-like state characterized by enhanced survival, phagocytosis, lipid metabolism, and tissue-compartmentalized responses around lesions. **(C)** Alzheimer’s disease: protective axis and therapeutic agonism. In AD models, reinforcing TREM2 signaling is proposed to augment DAM-associated clearance and trophic support, improving plaque containment/compaction, reducing secondary neuritic injury, and promoting neuroprotection. **(D)** Cancer: immunosuppressive TAM programs and therapeutic blockade. In multiple peripheral solid tumors, TREM2 marks TAMs with immunoregulatory properties; antagonistic or depleting anti-TREM2 strategies aim to dampen TREM2-driven suppressive circuits, threby enhancing antigen presentation and enabling more effective T-cell–mediated tumor control (often in combination with checkpoint blockade). **(E)** Peripheral TME logic. TREM2^+^ TAMs can shape an immune-excluded or T-cell–dysfunctional niche via soluble mediators (e.g., TGF-β, IL-10) and remodeling programs that collectively restrain cytotoxic CD8^+^ T-cell activity. **(F)** Emerging expression beyond canonical myeloid compartments. High-dimensional profiling has raised the possibility of context-restricted TREM2 expression in additional immune subsets in select disease settings; these observations remain under active investigation and require careful validation of cell-intrinsic versus myeloid-mediated effects. Overall, the figure emphasizes that tissue context and cellular identity can flip the therapeutic logic of TREM2 modulation—supporting agonism in some neurodegenerative settings and blockade in many peripheral cancers—while underscoring new frontiers in ligand biology and immune compartmentalization.

In addition to phagocytosis, TREM2 has been shown to regulate the polarization of macrophages (67). Upon activation, TREM2 induces the M2 macrophage phenotype, which is associated with anti-inflammatory cytokine production and tissue repair (67). This contrasts with the M1 phenotype, which promotes inflammation and is associated with disease progression. By promoting M2 polarization, TREM2 helps to resolve inflammation and support tissue repair processes, which is crucial not only for neurodegeneration but also in other conditions like rheumatoid arthritis and atherosclerosis. While TREM2 is canonically defined as a myeloid-restricted receptor, recent high-dimensional profiling suggests potential expression in other immune compartments under specific pathological conditions. Some studies indicate that TREM2 may be expressed by specific subsets of lipid-associated T cells or B cells in metabolic disorders and cancer, although these findings remain a subject of active investigation (68–72) (**Figure 3**). Understanding whether TREM2 exerts cell-intrinsic functions in lymphocytes or if these signals are secondary to myeloid interactions will be crucial for defining the full scope of TREM2 biology.

Recent studies have further elucidated the connection between TREM2 and neuroinflammation (73, 74). In Alzheimer’s disease, TREM2 mutations, particularly the R47H variant, impair the ability of microglia to clear amyloid plaques and regulate neuroinflammation (75, 76). These mutations lead to an increased risk of AD and are associated with an impaired immune response in the brain. Microglia in individuals with TREM2 mutations exhibit a reduced capacity to activate the PI3K/Akt pathway and are less efficient at clearing toxic debris, which contributes to the accumulation of amyloid plaques and the exacerbation of neuroinflammation (73, 77).

TREM2’s function is not limited to the CNS. In cancer, TREM2 has been shown to influence the tumor microenvironment by modulating the function of TAMs (62, 78–80). Similar to its role in NDDs, TREM2 in TAMs promotes an anti-inflammatory phenotype that helps tumors evade immune surveillance and promotes tumor progression. Interestingly, TREM2 overexpression in certain cancer models, such as GBM, has been shown to suppress tumor growth and synergize with immunotherapy, particularly PD-1 inhibitors, suggesting a dual role for TREM2 in both immune suppression and activation in cancer (3, 81).

The structure of TREM2 is highly consistent with its biological functions. The extracellular immunoglobulin-like domains are essential for its interaction with lipidated ligands, which is crucial for its immune-modulatory effects (82). The integrity of the extracellular domain is critical for ligand binding, and any mutations in this region can impair TREM2 function, as seen in Alzheimer’s disease. The intracellular signaling pathway, mediated by DAP12, ensures that the receptor’s activation translates into appropriate immune responses, such as macrophage polarization and phagocytosis (61). This structural-function relationship is particularly evident in the immune-regulatory roles of TREM2, where the receptor’s structural features allow it to detect signals from its environment and modulate immune cell behavior accordingly.

In conclusion, the structural design of TREM2 supports its pivotal role in immune regulation, particularly in the context of phagocytosis, macrophage polarization, and neuroinflammation. The consistency between its structural features and biological functions underpins its importance in both maintaining tissue homeostasis and regulating immune responses in diseases like Alzheimer’s, cancer, and autoimmune disorders. Ongoing research continues to unravel the full extent of TREM2’s biological functions and its therapeutic potential.

### TREM2 in Alzheimer’s disease

In recent years, TREM2 has gained significant attention due to its crucial role in the pathophysiology of Alzheimer’s disease (AD), particularly in modulating microglial function and influencing neuroinflammation. As the primary immune cells in the brain, microglia play an essential role in maintaining homeostasis, clearing cellular debris, and responding to injury or disease (83, 84). In AD, microglia become activated in response to amyloid-beta (Aβ) plaques and tau tangles, hallmarks of the disease (84–86). TREM2 has been shown to regulate the response of microglia to these pathological features, with significant implications for disease progression. Genetic studies have identified several rare missense mutations in the TREM2 gene that significantly increase susceptibility to late-onset AD and other neurodegenerative disorders. Among these, the R47H variant is the most strongly associated with AD risk, conferring a 2–4-fold increase in susceptibility (87, 88). This mutation disrupts ligand binding and reduces TREM2 surface expression, leading to impaired microglial activation and phagocytosis. Carriers of R47H exhibit diminished responses to amyloid-β (Aβ) aggregates and show increased plaque burden, neuroinflammation, and synaptic loss compared to non-carriers. Other variants, such as R62H and T66M, also compromise receptor stability and downstream DAP12-SYK signaling, reinforcing the concept that TREM2 loss-of-function mutations accelerate neurodegenerative pathology.

In the healthy brain, TREM2 plays a vital role in the activation of microglia, guiding their involvement in immune responses, phagocytosis, and synaptic pruning (31, 83). When microglia encounter Aβ plaques in Alzheimer’s disease, they attempt to clear the amyloid deposits through phagocytosis. TREM2 is thought to enhance this process, as well as to influence the activation and survival of microglia (89). TREM2-expressing microglia cluster tightly around Aβ plaques, facilitating plaque compaction and limiting the diffusion of toxic Aβ oligomers (90). This microglial barrier function is markedly reduced in TREM2-deficient or R47H mutant models, resulting in diffuse, less compacted plaques and heightened neuritic dystrophy. Functionally, TREM2 drives the transition of homeostatic microglia into disease-associated microglia, a phenotype specialized for lipid metabolism and debris clearance, through activation of the DAP12–SYK–PI3K–mTOR pathway.

**Figure 4:**
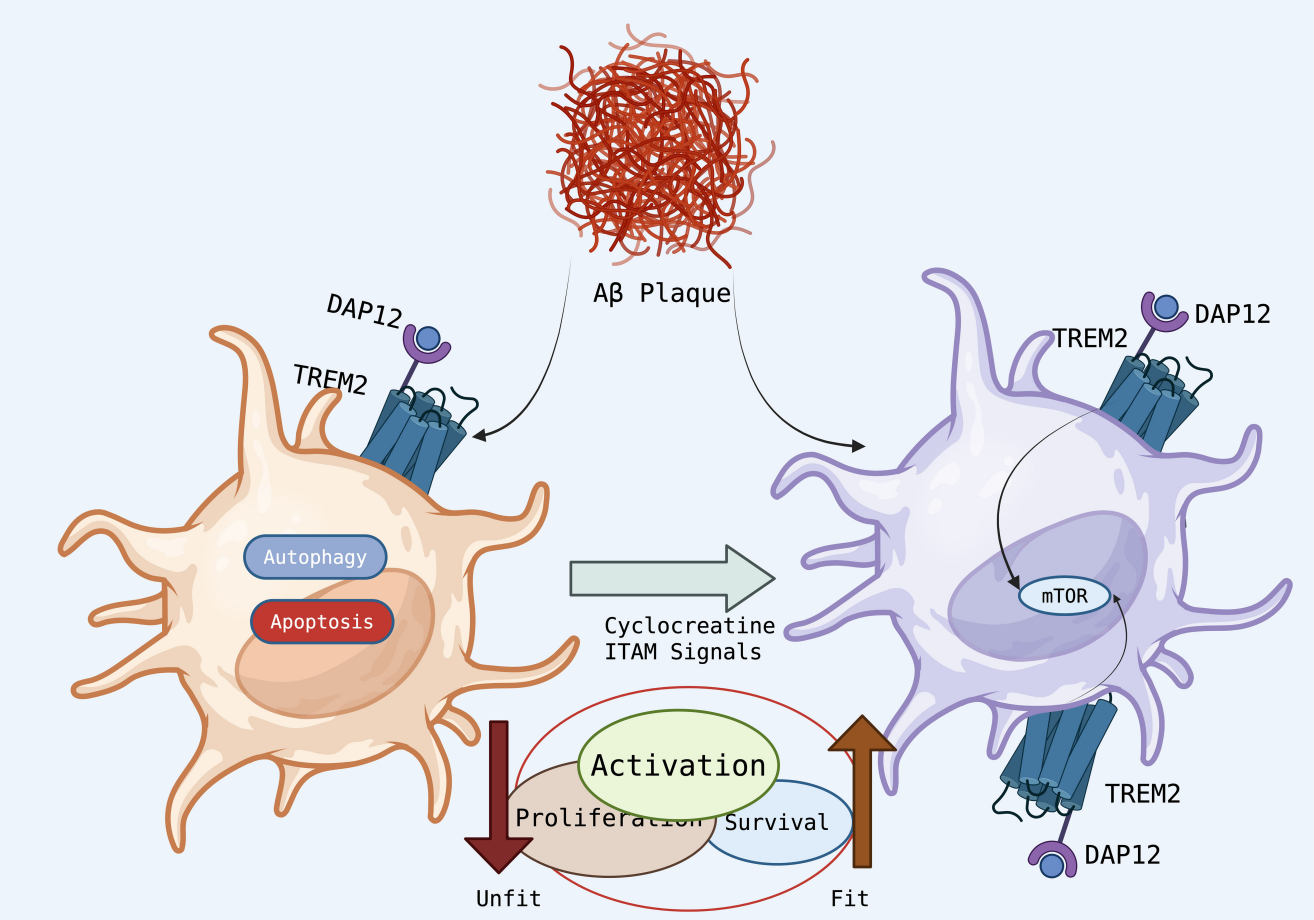
TREM2–DAP12 signaling enhances microglial fitness and Aβ handling via mTOR Model depicting how TREM2–DAP12 signaling promotes a metabolically “fit” microglial state in the context of amyloid-β (Aβ) plaques. Dysfunctional (“unfit”) microglia exhibit elevated markers of cellular stress (autophagy and apoptosis) and display reduced capacity for activation, proliferation, and survival. Note that TREM2 and DAP12 receptors are expressed on the cell surface but are functionally impaired. Right, ligand engagement of TREM2 couples to DAP12 ITAM signaling and activates downstream pathways including the mechanistic target of rapamycin (mTOR). This drives biosynthetic programs that increase microglial activation, proliferation, and survival, thereby improving the capacity to respond to and handle Aβ aggregates. The transition toward a fit phenotype is illustrated and can be potentiated by ITAM-supporting signals such as cyclocreatine. Cyclocreatine acts not as a direct receptor ligand, but essentially as a creatine analogue that supplements cellular ATP energy pools, providing the necessary metabolic foundation to sustain high-energy ITAM-dependent signaling cascades and restore microglial fitness. Aβ, amyloid-β; ATP, adenosine triphosphate; ITAM, immunoreceptor tyrosine-based activation motif; mTOR, mechanistic target of rapamycin.

One of the critical roles of TREM2 in Alzheimer’s disease is its regulation of neuroinflammation (91). Microglial activation in response to neurodegeneration often results in the release of pro-inflammatory cytokines, which contribute to the neuroinflammatory environment seen in AD (2). While microglia are typically protective in their early responses, prolonged activation and excessive inflammation can lead to neurotoxicity. TREM2 signaling has been shown to help modulate this response, promoting a protective phenotype of microglia that clears debris without causing excessive damage to surrounding neurons. In animal models of Alzheimer’s disease, the absence of functional TREM2 results in heightened neuroinflammation and an impaired ability to resolve inflammation effectively (28, 92). In contrast, overexpression of TREM2 has been associated with a more efficient microglial response, reduced neuroinflammation, and improved clearance of Aβ plaques (93). However, it is important to note that while TREM2 activation can suppress neuroinflammation, its exact role in the balance between neuroprotection and neurodegeneration is still a subject of ongoing research. Some studies suggest that chronic or excessive activation of TREM2 could contribute to persistent inflammation, thereby exacerbating neurodegeneration. **Figure 4** depicts this critical transition, showing how TREM2-DAP12 signaling activates mTOR to restore biosynthetic capacity. Importantly, this transition can be potentiated by alternative energy sources such as cyclocreatine. By supplementing the cellular ATP pool, cyclocreatine bypasses defective signaling nodes to fuel the high-energy demands of the ITAM-dependent ‘fit’ phenotype, thereby enhancing Aβ clearance even in the presence of partial receptor dysfunction.

### TREM2 as a therapeutic target in Alzheimer’s disease

AD therapies currently being explored focus on targeting the formation of amyloid-beta (Aβ) by modulating β-secretase and γ-secretase, activating α-secretase, or reducing Aβ accumulation through antibodies directed at Aβ monomers or aggregates (94–96). However, a deeper understanding of the role of TREM2 and its variants in microglia during the progression of AD pathology and NDDs is leading to the development of therapeutic approaches targeting TREM2. Since AD-associated TREM2 variants are known to exert loss-of-function effects, pharmacological strategies aimed at enhancing TREM2 expression, signaling, and activity could be beneficial, especially in patients carrying TREM2 variants or other genetic polymorphisms that impact microglial function. Strategies to increase TREM2 expression might involve inhibiting protease-mediated cleavage, while TREM2 signaling could be amplified through agonistic antibodies or small molecules that mimic phospholipid ligands. Additionally, since TREM2 supports microglial mTOR signaling and metabolism, metabolic agents that enhance microglial metabolic health may represent a viable therapeutic option.

Although some evidence suggests that boosting TREM2 activity in the early stages of AD, when Aβ plaques are just beginning to form, may be beneficial, other studies indicate that TREM2 expression in a tau model of AD may have detrimental effects during disease progression (97, 98). These findings remain contentious, as some research suggests that TREM2 expression in tau models could be beneficial (99). Until these conflicting results are clarified, it remains uncertain whether enhancing TREM2 activity throughout all stages of AD progression would be beneficial, or whether a strategy of boosting TREM2 in the early stages and inhibiting its activity in later stages of the disease might prove more effective (8).

Additionally, the development of biomarkers that assess TREM2 activity or expression could serve as a diagnostic tool, allowing for early detection of Alzheimer’s disease and more precise monitoring of therapeutic efficacy (100, 101). sTREM2 levels in CSF or blood have been proposed as potential biomarkers of microglial activation and neuroinflammation, though their reliability and clinical utility need further validation (102).

### TREM2 in cancer

#### TREM2 in tumor-associated myeloid cells

The tumor microenvironment is characterized by a heterogeneous mixture of immune and stromal cells, with TAMs being one of the most abundant and influential populations (103, 104). TAMs play a crucial role in tumor progression by regulating immune responses and influencing tumor growth, metastasis, and response to therapies. TREM2 has been shown to be preferentially expressed on TAMs and other myeloid-derived cells, where it acts as an immune regulator, modulating their activity in response to tissue damage or stress signals in the TME. The activation of TREM2 on these myeloid cells can lead to changes in their phenotype, affecting their pro- or anti-inflammatory properties (105).

In GBM and other primary brain tumors, TREM2 generally exhibits a neuroprotective and tumor-suppressive profile. Single-cell RNA-seq and spatial transcriptomic studies have shown that TREM2 is highly expressed in microglia-derived myeloid populations surrounding GBM lesions (3). In this context, TREM2 activation supports phagocytosis of necrotic and apoptotic cells, lipid clearance, and metabolic adaptation via DAP12–SYK–PI3K–mTOR signaling. Overexpression of TREM2 reprograms microglia toward an immune-competent phenotype, enhancing antigen presentation and cooperating with anti–PD-1 therapy to suppress tumor progression in murine GBM models (3). Conversely, TREM2 downregulation in glioma-infiltrating microglia is associated with impaired debris clearance, increased oxidative stress, and tumor proliferation (106). Thus, in the CNS context, TREM2 functions as an immunoprotective regulator maintaining local immune surveillance.

In contrast, studies in peripheral solid tumors—such as hepatocellular carcinoma, colorectal cancer, and melanoma—have revealed a different functional outcome. In these tumors, TREM2 is predominantly expressed on infiltrating TAMs derived from circulating monocytes rather than CNS-resident microglia. Here, TREM2 signaling promotes an immunosuppressive and pro-tumorigenic phenotype, characterized by increased IL-10 production, reduced antigen presentation, and metabolic reprogramming toward oxidative phosphorylation. Genetic depletion or antibody blockade of TREM2 in mouse models of melanoma and liver cancer enhances CD8^+^ T-cell infiltration and synergizes with PD-1 or CTLA-4 blockade, underscoring its role as a suppressor of anti-tumor immunity in non-CNS contexts.

This striking functional dichotomy—protective in the CNS versus deleterious in the periphery—appears to stem from fundamental differences in myeloid ontogeny and the specific metabolic constraints of TME. In the CNS, TREM2 serves as a critical sensor that sustains microglial metabolic fitness, enabling the phagocytosis of immunogenic debris and preventing the acquisition of an exhausted phenotype; thus, its activation reinvigorates anti-tumor surveillance (3, 106). In stark contrast, in peripheral solid tumors, TREM2 signaling in bone marrow-derived TAMs drives a specific immunosuppressive transcriptional program, often characterized by the metabolic sequestration of nutrients and the secretion of factors that actively exclude cytotoxic CD8+ T cells (12, 107). Therefore, TREM2 acts as a ‘double-edged sword’ in oncology: it functions as a context-dependent immune switch that can either support tumor containment or fuel immune evasion, necessitating diametrically opposing therapeutic strategies (agonism vs. antagonism) depending on the anatomical location of the malignancy. This functional dichotomy constitutes a central paradox in TREM2 biology. As illustrated in **Figure 3**, the tissue context acts as a determinant switch: in the Alzheimer’s disease microenvironment (**Figure 3C**), TREM2 signaling is essential for metabolic fitness and plaque containment, representing a protective ‘gain-of-function’ mechanism. In sharp contrast, within the peripheral tumor microenvironment (**Figure 3E**), the same signaling machinery drives an immunosuppressive, nutrient-hoarding macrophage phenotype that excludes T cells. This visual comparison underscores that therapeutic strategies must be diametrically opposed—agonism for neurodegeneration versus antagonism for peripheral cancer—depending on the specific metabolic niche.

#### Mechanisms of TREM2 signaling in cancer

The signaling pathways downstream of TREM2 are critical in determining its role in the TME. As described in the ‘Biology of TREM2’ section, TREM2 signaling in TME relies on the canonical DAP12-SYK and DAP10-PI3K axes. However, the functional outcome of this signaling in TAMs is often distinct from that in microglia, driving an immunosuppressive rather than protective phenotype (**Figure 3**). In cancers, the ligands of TREM2 are thought to include various damage-associated molecular patterns (DAMPs) and lipids that are released from dying tumor cells, necrotic tissue, or damaged extracellular matrix components (29). One of the key classes of TREM2 ligands in cancer is sphingolipids, which are enriched in the CNS and have been shown to bind TREM2 on myeloid cells, particularly in GBM (29). The interaction between TREM2 and sphingolipids in the TME has been shown to activate a protective signaling response in TAMs, leading to a shift toward a more anti-inflammatory phenotype (108). This reprogramming of TAMs by TREM2 ligands may contribute to the immunoprotective role of TREM2 in CNS cancers, as it enhances the phagocytic activity of myeloid cells and promotes the clearance of tumor debris. However, in peripheral cancers, the same pathways may drive the immunosuppressive polarization of TAMs, depending on the presence of specific ligands and the overall composition of the TME​.

#### Therapeutic targeting of TREM2 in cancer

**Figure 5:**
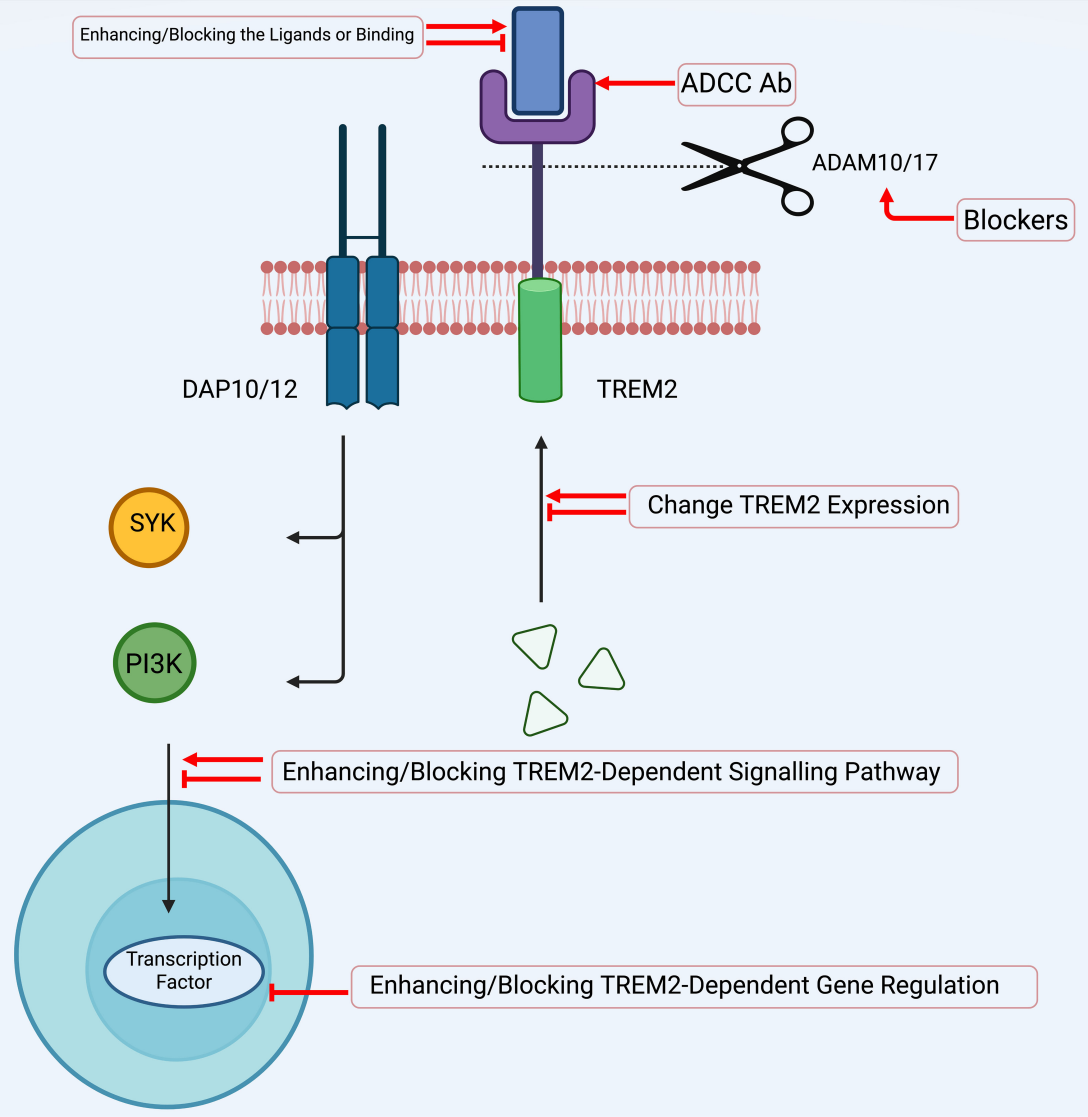
Therapeutic entry points for modulating TREM2 signaling. Extracellular interventions include (i) ligand-level modulation—enhancing or blocking ligand availability/affinity to alter receptor engagement; (ii) agonistic/ADCC-capable antibodies that cluster TREM2 to drive signaling or deplete targeted cells; and (iii) sheddase control, where inhibition of ADAM10/17 reduces ectodomain shedding to preserve surface TREM2. At the receptor/cell level, expression modifiers (e.g., small molecules, gene therapy) increase or decrease TREM2 abundance. Intracellularly, pathway-directed agents can enhance or inhibit DAP10/12–SYK–PI3K cascades, reshaping signal strength and duration. These inputs converge on transcription-factor programs to augment or suppress TREM2-dependent gene regulation, ultimately affecting microglial activation states and effector functions. ADAM, A Disintegrin and Metalloproteinase; ADCC, antibody-dependent cellular cytotoxicity; SYK, spleen tyrosine kinase; PI3K, phosphatidylinositol 3-kinase.

Given its critical role in regulating the immune response in cancer, TREM2 has emerged as a potential therapeutic target for cancer immunotherapy. Several strategies are being explored to modulate TREM2 activity in the TME, including the use of TREM2 agonists, antagonists, and immune checkpoint inhibitors (109–111). In particular, the combination of TREM2-targeting therapies with immune checkpoint blockade (ICB) has shown promise in preclinical cancer models (106, 112). By enhancing the activation of TREM2 in TAMs, it may be possible to reprogram the TME from an immunosuppressive state to a more immune-stimulatory one, thus improving the efficacy of existing immunotherapies (**Figure 5**).

For example, in GBM, where TREM2 is downregulated in TAMs during tumor progression, restoring TREM2 expression using adeno-associated virus (AAV)-mediated gene therapy has been shown to suppress tumor growth and enhance the antitumor immune response (113). This approach synergizes with anti-PD-1 therapy, highlighting the potential of combining TREM2-targeting strategies with ICB to overcome the immune evasion mechanisms employed by tumors (3, 113)​.

### TREM2 in other diseases

#### TREM2 and other NDDs

While the majority of research on TREM2 has focused on its role in Alzheimer’s disease, recent studies suggest that TREM2 may also play a role in other NDDs, including Parkinson’s disease (PD), frontotemporal dementia (FTD), and amyotrophic lateral sclerosis (ALS) (114–116). In these diseases, microglia are also implicated in disease pathogenesis, and mutations in TREM2 have been linked to increased susceptibility to these conditions. However, the exact role of TREM2 in these diseases is less well understood, and further research is needed to determine whether therapeutic strategies targeting TREM2 could be beneficial across a broader spectrum of NDDs.

#### TREM2 and PD

Recent research has highlighted the critical role of TREM2 in the pathogenesis of PD. Clinical studies have reported a significant correlation between CSF sTREM2 levels and α-synuclein in PD patients, suggesting a link between microglial activation and disease pathology (41, 114, 117–119). Furthermore, mechanistic studies in animal models indicate that TREM2 deficiency may impair the microglial capacity to internalize and degrade α-synuclein aggregates, thereby exacerbating neuroinflammation (119). While genetic variants like R47H are more strongly linked to Alzheimer’s disease, emerging evidence suggests TREM2 polymorphisms may also influence PD susceptibility by altering microglial function and lipid metabolism, which are crucial for neuronal health (120, 121). Therapeutic strategies targeting TREM2, such as agonists or gene therapy, show promise in preclinical models by enhancing toxic aggregate clearance and reducing neuroinflammation. However, the exact mechanisms of TREM2 in PD remain unclear, necessitating further research to explore its role across disease stages and its potential as a therapeutic target.

#### TREM2 and FTD

TREM2 has also been implicated in the pathogenesis of FTD, a neurodegenerative disorder characterized by progressive damage to the frontal and temporal lobes. In FTD, dysfunctional TREM2 signaling has been associated with impaired microglial activity, leading to inadequate clearance of pathological protein aggregates, such as TDP-43, which are commonly observed in FTD patients (2, 8). Genetic variants of TREM2, particularly the R47H mutation, have been identified as risk factors for FTD, highlighting the importance of TREM2 in maintaining brain homeostasis and modulating neuroinflammation (88).

Research suggests that TREM2 dysfunction exacerbates neuroinflammation and contributes to neuronal loss in FTD (122, 123). Impaired TREM2 activity disrupts microglial responses to neurodegeneration, leading to chronic inflammation and accelerated disease progression. Additionally, TREM2’s role in lipid metabolism is thought to be relevant in FTD, as lipid dysregulation has been observed in affected brain regions. Therapeutic strategies aimed at enhancing TREM2 function, such as TREM2 agonists or gene therapy, are being explored in preclinical models to restore microglial function, reduce inflammation, and promote neuronal survival (124). However, further studies are needed to fully understand the mechanisms by which TREM2 contributes to FTD pathology and to evaluate the potential of TREM2-targeted therapies in clinical settings. In summary, TREM2 represents a promising therapeutic target for FTD, with the potential to modulate microglial activity and mitigate disease progression.

#### TREM2 and ALS

The role of TREM2 in ALS is an area of growing interest, particularly as it relates to microglial function, neuroinflammation, and neurodegeneration. Canonical TREM2 undergoes proteolytic processing to generate sTREM2, which has been implicated in microglial activation and survival (125). In ALS, elevated levels of sTREM2 in the CSF have been observed, particularly in early disease stages, and higher sTREM2 levels correlate with slower disease progression, suggesting a protective role (126). Future studies should focus on identifying the ligands and receptors for sTREM2 in ALS, as well as its potential role in clearing pathological proteins like TDP-43 and apoptotic neurons (127, 128). Additionally, the spatial-temporal dynamics of DAM in ALS remain poorly understood. DAM, which exhibit a high phagocytic capacity, may serve as sensors for neurodegeneration-associated molecular patterns, such as dying neurons, myelin debris, and protein aggregates (129). Investigating the triggers for DAM phenotype induction and the role of TREM2 in this process will be critical. Furthermore, TREM2-dependent regulation of synaptic pruning and microglia-neuron interactions may play a role in modulating neuronal hyperexcitability, a hallmark of ALS (130). Understanding how TREM2 influences these interactions at different disease stages and in various ALS pathologies will be essential for developing targeted therapies.

#### TREM2 and cerebrovascular diseases

TREM2 plays a critical role in regulating neuroinflammation and supporting neuroprotection in cerebrovascular diseases (CVDs), particularly in ischemic stroke (131, 132). TREM2 activation through various therapeutic approaches, such as electroacupuncture and ligand interactions, has been shown to enhance neuroprotection and improve recovery after ischemic stroke research directions, targeting TREM2 for therapeutic intervention in CVDs holds significant promise (133). Ongoing studies are investigating TREM2 as a biomarker for stroke prognosis, where sTREM2 levels correlate with stroke severity and long-term outcomes (18). Furthermore, future research should focus on fully elucidating the mechanisms by which TREM2 modulates microglial activity and neuroinflammation in CVDs. Understanding the specific signaling pathways involved, such as the PI3K/Akt pathway, and exploring combination therapies with other anti-inflammatory agents could lead to novel treatment strategies aimed at enhancing recovery and reducing neurological deficits in patients post-stroke (134).

## Challenges and future directions

TREM2 has emerged as a pivotal modulator of immune responses, influencing a range of diseases including neurodegenerative conditions, cancer, autoimmune disorders, and cerebrovascular diseases (5, 18, 23, 135–138). Despite the growing recognition of its critical role, harnessing therapeutic potential of TREM2 remains fraught with challenges, particularly due to its dualistic nature and the complexity of its signaling pathways. As research continues to illuminate the various facets of TREM2 biology, several key challenges and future research directions must be considered to fully exploit its therapeutic benefits across diverse disease contexts.

### Targeting TREM2 in NDDs

The role of TREM2 in NDDs such as AD and PD is well documented, with mutations in TREM2 associated with impaired immune responses, increased neuroinflammation, and defective clearance of toxic substances. In particular, loss-of-function variants such as R47H diminish microglial responsiveness to amyloid-β (Aβ), impair plaque compaction, and exacerbate neurodegeneration (139). This genetic and functional evidence provides a strong rationale for TREM2 agonism in AD: restoring or enhancing TREM2 signaling may help normalize microglial function and promote the clearance of protein aggregates and lipid debris.

In the context of Alzheimer’s disease, therapeutic strategies primarily focus on TREM2 agonism. Translating TREM2 agonism into the clinic has revealed significant hurdles regarding efficacy and delivery. AL002 (Alector/AbbVie), a humanized IgG1 agonist, advanced to the Phase 2 INVOKE-2 trial in early Alzheimer’s disease. Despite promising Phase 1 safety data (109), top-line results released in late 2024 indicated that AL002 failed to meet its primary endpoint (CDR-SB) and was associated with higher rates of ARIA-like imaging abnormalities in APOE ϵ4 carriers, leading to the discontinuation of its extension study (140). This setback highlights the challenge of balancing receptor activation with potential inflammatory side effects. While murine models have been instrumental in elucidating the downstream signaling of TREM2, a critical gap remains between these models and human genetics. For instance, the R47H variant significantly increases AD risk in humans, but many transgenic mouse models fail to fully recapitulate the late-onset neurodegenerative features or the exact plaque-associated microglial dynamics seen in human carriers (59, 141). This discrepancy may stem from species-specific differences in the microglial transcriptome and the diverse lipidome of the human brain (142, 143). Future research must prioritize ‘humanized’ TREM2 models and integrate longitudinal human PET imaging data to validate whether the pro-survival effects observed in rodents truly translate to clinical neuroprotection.

In parallel, *Iluzanebart* (VGL101, Vigil Neuroscience), targeting the rare microgliopathy ALSP, has demonstrated durable target engagement and safety in the Phase 2 IGNITE trial, serving as a proof-of-concept for TREM2 agonism in genetic leukodystrophies (144). A major pharmacological barrier remains BBB penetration. To address this, DNL919 (Denali) utilized an Antibody Transport Vehicle binding the transferrin receptor to enhance CNS uptake; however, its development was halted due to peripheral hematologic safety signals (140, 145). Collectively, these trials underscore that successful TREM2 therapy requires precise optimization of BBB delivery and rigorous safety monitoring to avoid off-target immune activation.

Pharmacodynamic readouts from some programs include transient increases in CSF sTREM2, shifts in microglial-related biomarkers, and modulation of neuroinflammatory signatures, consistent with *in vivo* activation of TREM2-dependent pathways (146). However, it remains unclear whether sustained TREM2 agonism will translate into clinically meaningful benefits on cognition or long-term disease progression. Key challenges include efficient blood–brain barrier penetration, optimal dosing to avoid over-activation of microglia, and the possibility that the therapeutic window is restricted to early disease stages before extensive neuronal loss and tau pathology have occurred.

At the same time, therapeutic strategies aimed at modulating TREM2 activity must reckon with the functional variability introduced by different TREM2 variants and disease contexts. While boosting TREM2 activity in carriers of loss-of-function mutations such as R47H may be beneficial, excessive or prolonged activation could theoretically drive chronic inflammation or exacerbate other pathologies. Future approaches are therefore likely to focus on stage-specific and context-dependent enhancement of TREM2 signaling, particularly in early-stage neurodegeneration, where its activation might facilitate the clearance of toxic proteins such as Aβ and tau.

While the rationale for TREM2 agonism in amyloid-driven pathology is relatively strong, its role in tauopathies remains a subject of intense debate and biological complexity. The relationship between TREM2 and Tau is not straightforward and appears to be strictly context-dependent. On one hand, studies utilizing Trem2-deficient models have demonstrated that loss of TREM2 signaling exacerbates tau pathology, suggesting that functional microglia are required to phagocytose and degrade pathological tau seeds (99, 147). In this ‘protective’ model, TREM2 activation would be beneficial by restraining the spatial spread of tau aggregates. Conversely, conflicting findings suggest that TREM2 activation may inadvertently fuel neurodegeneration in the presence of established tauopathy. In certain P301S tau transgenic models, TREM2 deficiency was surprisingly found to be protective, reducing brain atrophy and preserving excitatory synapses (148). This ‘detrimental’ effect is hypothesized to stem from the fact that activated microglia may secrete neurotoxic factors or physically facilitate the trans-synaptic propagation of tau seeds via exosomal release. Thus, unlike in AD where early activation is universally favored, the therapeutic window for TREM2 in primary tauopathies may be much narrower. It is plausible that TREM2 agonism is beneficial during the early ‘seeding’ phase but potentially harmful once widespread inflammation and neuronal stress are established. Resolving this dichotomy—determining when to step on the gas and when to hit the brake —remains a critical prerequisite for clinical translation.

### Expanding TREM2’s role in cancer immunotherapy

The emerging role of TREM2 in cancer, particularly in regulating TAMs, adds another layer of complexity to its biology. TREM2 is a key regulator of macrophage polarization and has been implicated in tumor progression, immune evasion, and responses to immunotherapy. In peripheral solid tumors such as melanoma, colorectal cancer, and hepatocellular carcinoma, TREM2 is predominantly expressed on immunosuppressive, metabolically adapted TAMs (72, 138, 149). Conversely, in peripheral solid tumors, the goal is often to inhibit TREM2-mediated immunosuppression. Here, antagonistic or blocking antibodies are employed to disrupt the maintenance of immunosuppressive TAMs, thereby unleashing T-cell responses and enhancing the efficacy of immune checkpoint inhibitors (12). This strategy functions as a myeloid checkpoint blockade, reducing the number of immunosuppressive TREM2+ TAMs and promoting anti-tumor immunity (12, 107). Combination regimens of anti-TREM2 with immune checkpoint inhibitors such as anti-PD-1 or anti-CTLA-4 have shown additive or synergistic effects in several preclinical models, further supporting the concept that TREM2 can be therapeutically relieved to reprogram the myeloid compartment and potentiate immunotherapy (79, 112, 150, 151). In this context, TREM2-targeting antibodies generally act as antagonists or depleting agents, in clear contrast to the agonistic designs being explored in Alzheimer’s disease.

By contrast, in GBM and other CNS tumors, the functional outcome of TREM2 signaling appears to be different and more context-dependent. Here, TREM2 is expressed mainly in microglia-dominated myeloid populations, and recent studies suggest that it may exert an immunoprotective rather than purely immunosuppressive role. In murine GBM models, genetic deletion or pharmacological inhibition of TREM2 accelerates tumor growth, skews TAMs toward a more immunosuppressive Arg1^+^ phenotype, and aggravates T-cell exhaustion, whereas experimental overexpression or AAV-mediated delivery of TREM2 to CNS myeloid cells restrains tumor progression and enhances the efficacy of anti-PD-1 therapy (152). Thus, in the brain tumor setting, TREM2 upregulation may improve the responsiveness to immune checkpoint blockade, and TREM2 agonism or restoration, rather than blockade, could be beneficial. These observations indicate that TREM2 can act as both an immune-suppressive factor and a therapeutic target, with its net effect determined by the tissue, cell lineage, and tumor context.

However, targeting TREM2 in cancer presents substantial challenges. The marked heterogeneity of TAMs across different tumor types and anatomical sites implies that TREM2’s role may vary widely, and modulation of its activity could have unintended consequences, including treatment resistance, excessive inflammation, or off-target immune effects. The same intervention that is beneficial in peripheral tumors could be detrimental in CNS malignancies, or vice versa. In addition, the use of TREM2 agonists or antagonists will likely require precise delivery strategies to selectively target tumor-associated myeloid cells while sparing homeostatic myeloid populations in other organs. Future research should aim to refine our understanding of TREM2’s context-dependent functions within the tumor microenvironment, delineate how it intersects with other immune checkpoints and metabolic pathways, and define biomarker-guided approaches to identify patients and tumor types most likely to benefit from TREM2-directed therapies.

### The need for specificity in therapeutic targeting

A major challenge in TREM2-based therapies is ensuring the specificity of treatment. TREM2 is involved in various cellular processes that are crucial for immune responses, including phagocytosis, cytokine release, and cell survival​. Therefore, manipulating TREM2 activity could lead to unintended effects on immune homeostasis or tissue repair. The specificity of TREM2-targeting therapies must be ensured through the development of selective agonists or antagonists that can modulate its activity without causing excessive inflammation or immune suppression (62, 89).

Furthermore, TREM2’s involvement in various diseases necessitates the development of disease-specific strategies. In NDDs, for example, TREM2-targeting approaches might focus on enhancing its ability to clear toxic debris, while in cancer, the focus may shift towards modulating the immune microenvironment. Understanding these differential roles will be crucial in designing precision therapies that can adapt TREM2’s effects to the specific needs of the disease context​.

While TREM2 holds significant promise as a therapeutic target in a variety of diseases, including neurodegeneration, cancer, and autoimmune conditions, there are substantial challenges in fully harnessing its potential (10). These include the complexity of its signaling, the dual roles it plays in different disease contexts, and the need for specificity in therapeutic interventions. Future research should prioritize elucidating the molecular mechanisms underlying TREM2’s diverse functions and exploring context-dependent strategies for targeting this receptor. Through these efforts, we can fully harness the therapeutic potential of TREM2, providing novel and promising avenues for the treatment of patients afflicted with a spectrum of debilitating diseases.

## Conclusion

TREM2 holds significant potential as a therapeutic target across a wide range of diseases, including neurodegenerative disorders, cancer, and autoimmune conditions. However, its complex and context-dependent role in immune modulation presents several challenges. The dual nature of effects of TREM2, where it can be both protective and detrimental depending on the disease environment, complicates its therapeutic application. Future research should focus on elucidating the specific mechanisms underlying TREM2 signaling, its interactions with other immune pathways, and how it modulates immune responses across different diseases (153). Additionally, developing more precise and targeted therapeutic strategies, such as specific TREM2 agonists or antagonists, will be essential to ensure its safe and effective use in clinical settings. Understanding the potential of TREM2 as a biomarker for disease progression and treatment response will also be crucial in advancing personalized therapies (153). Ultimately, overcoming these challenges will unlock TREM2’s therapeutic potential and offer new avenues for treatment in various diseases, providing hope for improved patient outcomes.
